# Syphilis in pregnancy detection rate: time series study associated with descriptive analysis by periods, Campo Grande, 2015–2023

**DOI:** 10.1590/S2237-96222026v35e20250940.en

**Published:** 2026-07-31

**Authors:** Caroliny Oviedo Fernandes Reis, Luciana Aparecida da Cunha Borges, Cláudia Du Bocage Santos-Pinto, Ana Lúcia Lyrio de Oliveira, Daniele Soares-Marangoni, Carlos Dornels Souza Freire, Everton Falcão de Oliveira

**Affiliations:** 1 Universidade Federal de Mato Grosso do Sul Programa de Pós-Graduação em Doenças Infecciosas e Parasitárias Campo Grande MS Brazil Universidade Federal de Mato Grosso do Sul, Programa de Pós-Graduação em Doenças Infecciosas e Parasitárias, Campo Grande, MS, Brazil; 2 Universidade Federal de Mato Grosso do Sul Faculdade de Medicina Campo Grande MS Brazil Universidade Federal de Mato Grosso do Sul, Faculdade de Medicina, Campo Grande, MS, Brazil; 3 Universidade Federal de Mato Grosso do Sul Instituto Integrado de Saúde Campo Grande MS Brazil Universidade Federal de Mato Grosso do Sul, Instituto Integrado de Saúde, Campo Grande, MS, Brazil; 4 Universidade Federal do Vale do São Francisco Faculdade de Medicina Petrolina PE Brazil Universidade Federal do Vale do São Francisco, Faculdade de Medicina, Petrolina, PE, Brazil

**Keywords:** Syphilis, Pregnancy, Disease Notification, Health Information Systems, Time Series Studies, Sífilis, Embarazo, Notificación de Enfermedades, Sistemas de Información en Salud, Estudios de Series Temporales

## Abstract

**Objective:**

To analyze the time series and trend of the syphilis in pregnancy detection rate over nine years and the variations in clinical and epidemiological characteristics across four periods according to relevant public health events in Campo Grande, Mato Grosso do Sul.

**Methods:**

Time series study with descriptive analysis by periods, using data from the Notifiable Diseases Information System (*Sistema de Informação de Agravos de Notificação*, SINAN) and the Live Birth Information System (*Sistema Nacional de Nascidos Vivos*, SINASC). Segmented linear regression was used to analyze the temporal trend of the syphilis in pregnancy detection rate, and the Kruskal-Wallis test was employed to analyze clinical and epidemiological variables across the evaluated periods: penicillin shortage (2015–2016); supply normalization and pre-pandemic period (2017–2019); pandemic (2020–2021); and post-COVID-19 pandemic (2022–2023).

**Results:**

A total of 2,802 cases were analyzed. An increase of 170.4% in the detection rate was observed, with a rising trend until 2018 (annual percentage change, APC 31.18%; 95% confidence interval, 95%CI 4.79; 118.80; p-value 0.017) and subsequent stabilization (APC 1.16%; 95%CI -27.24; 15.83; p-value 0.936). Regarding clinical-epidemiological characteristics, there was an increase in the frequency of latent phase, reactive treponemal tests, adequate treatment, and maternal education, and a reduction in partner treatment. During 2017–2019, there was a reduction in the use of non-treponemal tests; in 2020–2021, a decrease in Brown (Brazilian mixed race)/Black/Asian/Indigenous women; and in 2022–2023, an increase in prenatal care visits and late diagnoses.

**Conclusion:**

The syphilis in pregnancy detection rate increased until 2018 and remained stable thereafter. Changes in clinical and epidemiological characteristics reflect variations in the care context, with advances in prenatal care, but persistence of weaknesses in the timely diagnosis and treatment of partners.

## Resumen

**Objetivo:**

Analizar la serie temporal y la tendencia de la tasa de detección de sífilis durante el embarazo durante nueve años, así como las variaciones de las características clínico-epidemiológicas en cuatro períodos, según eventos sanitarios relevantes en salud pública en Campo Grande, Mato Grosso do Sul.

**Métodos:**

Estudio de serie temporal con análisis descriptivo por períodos, utilizando datos del Sistema de Información de Enfermedades de Notificación Obligatoria (*Sistema de Informação de Agravos de Notificação*, SINAN) y del Sistema de Información sobre Nacidos Vivos (*Sistema Nacional de Nascidos Vivos*, SINASC). La regresión lineal segmentada se utilizó para analizar la tendencia temporal de la tasa de detección de la sífilis durante el embarazo. La prueba de Kruskal-Wallis se utilizó para analizar las variables clínico-epidemiológicas entre los períodos evaluados: desabastecimiento de penicilina (2015–2016); normalización del abastecimiento y período prepandémico (2017–2019); pandemia (2020–2021) y pospandemia de COVID-19 (2022–2023).

**Resultados:**

Se analizaron 2.802 casos. Se observó un aumento del 170,4% en la tasa de detección, con tendencia creciente hasta 2018 (variación porcentual anual, VPA 31,18%; intervalo de confianza del 95%, IC95% 4,79; 118,80; p-valor 0,017) y estabilización posterior (VPA 1,16%; IC95% -27,24; 15,83; p-valor 0,936). En las características clínico-epidemiológicas, se verificó un aumento de la frecuencia de la fase latente, de la positividad de las pruebas treponémicas, del tratamiento adecuado y de la escolaridad de la mujer, y una reducción del tratamiento de las parejas. En el período 2017–2019, hubo una reducción en el uso de pruebas no treponémicas; en 2020–2021, una reducción en el uso por parte de mujeres mestizas (pardas)/negras/amarillas/indígenas; y en 2022–2023, un aumento en las consultas de control prenatal y en los diagnósticos tardíos.

**Conclusión:**

La tasa de detección de sífilis durante el embarazo aumentó hasta 2018 y se mantuvo estable posteriormente. Los cambios en las características clínicas y epidemiológicas reflejan variaciones en el contexto asistencial, con avances en el cuidado prenatal, pero la persistencia de fragilidades en el diagnóstico oportuno y en el tratamiento de las parejas.

**Ethical aspects**

This research respected ethical principles, having obtained the following approval data:

Research ethics committee Universidade Federal de Mato Grosso do Sul

Opinion number 5,080,108

Approval date 4/11/2021

Certificate of submission for ethical appraisal 52447421.0.0000.0021

Informed consent form Exempt.

## Introduction

Syphilis, a sexually transmitted infection caused by *Treponema pallidum*, is considered a global public health problem, with a continuous increase in cases since the 2000s, despite the availability of effective treatment [1]. Vertically transmitted syphilis contributes to miscarriages, stillbirths, and neonatal deaths, in addition to increasing the risk of low birth weight, prematurity, and the possibility of irreversible damage such as deafness, bone alterations, and neurological impairments [1-5]. 

In 2016, an estimated 988,000 probable cases of active syphilis occurred in pregnant women worldwide, with the highest burden in Africa and relevant frequency in the Americas. Of these, 661,000 were cases of congenital syphilis, of which 54% presented adverse events, including fetal and stillbirth deaths (40%), clinical manifestations of congenital syphilis (31%), neonatal death (17%), and prematurity or low birth weight (12%) [1]. In Brazil, from 2013 to 2024, there was an increase of 295% in the syphilis in pregnancy detection rate, 74% in the incidence of congenital syphilis, and 33% in infant mortality specifically due to congenital syphilis [6].

There were relevant public health events that may have compromised syphilis control and prevention strategies. One example is the worldwide penicillin shortage from 2014 to 2016 [7,8]. This negatively impacted the treatment of syphilis in pregnant women, which is a cornerstone for preventing vertical transmission [1,9]. Between 2014 and 2018, Brazil recorded an increase of 65% in the incidence rate of congenital syphilis and 43% in syphilis-related deaths among children under one year of age [6]. Another event of public health relevance was the COVID-19 [10,11] pandemic. Measures such as social distancing and reduced availability of essential services, including public transportation and health services, decreased the demand for sexual and reproductive health services, with a direct impact on timely diagnosis and treatment [10-13]. After the pandemic, health services faced accumulated demands and worsening of preventable conditions due to prolonged lack of care [14].

With the increasing cases of syphilis in pregnant women, studying temporal trends becomes fundamental to describe the disease evolution over time and identify periods with possible weaknesses in the healthcare response. However, uncertainties remain regarding how relevant public health events, such as penicillin shortages and the COVID-19 pandemic, were associated with changes in the clinical-epidemiological characteristics of syphilis in pregnancy. 

This study aimed to analyze the time series and trend of the syphilis in pregnancy detection rate over nine years, as well as to examine the frequencies of clinical and epidemiological characteristics of the disease across four periods defined by relevant public health events in Campo Grande, Mato Grosso do Sul.

## Methods

### Study design

This is a time series study with descriptive analysis by period, which observed the behavior of syphilis in pregnancy detection rate and the clinical and epidemiological variations over four periods from 2015 to 2023 in Campo Grande, Mato Grosso do Sul. The periods were defined according to the occurrence of public health events, identified as possible factors aggravating certain health conditions, including syphilis.

- Period P1 (2015–2016): worldwide penicillin shortage, with 60.7% of Brazilian states reporting scarcity [15].
- Period P2 (2017–2019): progressive normalization of supply in Brazil, before the COVID-19 pandemic [7,8,11].
- Period P3 (2020–2021): COVID-19 pandemic, with interruptions in routine healthcare service delivery [11].
- Period P4 (2022–2023): post-pandemic, with gradual resumption of routine healthcare services and programs [14].

### Setting

Campo Grande, the capital of Mato Grosso do Sul, is located in Brazil’s Central-West region and, by 2022, it had approximately 898,000 inhabitants [16]. Of these, 271,901 are women of reproductive age [16]. In the municipality, an average of 13,412.8 live births were recorded between 2015 and 2023 [17]. 

### Participants

All cases of syphilis in pregnancy reported to the Notifiable Diseases Information System (*Sistema de Informação de Agravos de Notificação*, SINAN) from 2015 to 2023 were considered eligible. Duplicate cases and non-treponemal test titers≤1:4 were excluded, as according to the Ministry of Health guidelines, these indicate serological scar in the absence of clinical or epidemiological evidence of active infection. Data on the newborn child were also obtained from the Live Birth Information System (*Sistema Nacional de Nascidos Vivos*, SINASC).

### Variables

The following variables were extracted from SINAN: age (continuous, in years), age group (10–19; 20–29; 30–39; ≥40 years), race/skin color (White; Brown [Brazilian mixed race]/Black/Asian/Indigenous; not informed), years of schooling (≤9 years; 10–12 years; ≥13 years; not informed), occupation (yes; no; not informed), gestational age at diagnosis (first; second; third; not informed), clinical classification of syphilis (primary; secondary; tertiary; latent; not informed), treponemal tests (reactive; non-reactive; not performed; not informed) and non-treponemal tests (reactive; non-reactive; not performed; not informed) during prenatal care, non-treponemal titers (1:8 to 1:32; <1:32 and ≤1:64; ≥1:128), treatment prescribed to the patient (with penicillin; without penicillin; not performed; not informed), adequacy of maternal treatment (adequate; inadequate; not informed), partner treatment (not performed; with penicillin; without penicillin; not informed), and reason untreated partner (no further contact; not notified or summoned to the health unit; did not attend for treatment; refused treatment; non-reactive serology; other reason). 

Race/skin color was self-reported individually at the time of notification, but, for this study, was grouped into three categories as described above. For the variable “adequacy of treatment,” the dose recommended by the Ministry of Health for each clinical stage of syphilis according to the year was considered [9,18]. 

From SINASC, the following variables were used: marital status (single; married or stable union; separated or divorced; not informed), number of visits (none; 1–5; 6 or more; not informed), start of prenatal care (early; late; not informed), Apgar score at 1 and 5 minutes (no asphyxia; moderate asphyxia; severe asphyxia; not informed), gestational age (preterm; term; postterm; not informed), birth weight (low; normal; macrosomia; not informed), and presence of congenital anomalies (no; yes; not informed).

### Data sources and measurement

The Municipal Health Department of Campo Grande provided nominal data from SINAN and SINASC. In the absence of non-treponemic titers in SINAN notifications, these results were searched in the electronic system of the Institute for Research, Teaching and Diagnostics of the Association of Parents and Friends of Exceptional Children of Campo Grande (*Instituto de Pesquisas, Ensino e Diagnósticos da Associação de Pais e Amigos dos Excepcionais de Campo Grande*, IPED/APAE). This is a clinical laboratory responsible for prenatal screening of pregnant women attended by the Brazilian Unified Health System (*Sistema Único de Saúde*, SUS) and partner institutions in Mato Grosso do Sul. The search was conducted using the patient’s name, date of birth, and mother’s name. A maximum retrospective interval was considered on the gestational trimester recorded in SINAN, measured from the notification date: up to 10 months for the first trimester, up to 7 months for the second, and up to 4 months for the third. These intervals covered the expected period for routine laboratory testing during prenatal care, based on the gestational age at the time of notification.

Next, duplicate records were identified and excluded. Duplicates were defined as repeated notifications of syphilis in pregnancy from the same disease episode for the same patient. In identifying duplicates, the diagnosis date, reported gestational trimester, and non-treponemal titer were considered (Supplementary Table 1). 

**Table 1:** Temporal trend of the detection rate of syphilis in pregnancy per 1,000 live births, annual percent change (APC), mean percent change (MPC), and 95% confidence interval (95%CI). Campo Grande, 2015–2023 (n=2,802)

Period	n (%)	MPC	APV	95%CI	p-value	Trend
2015–2023	2,802 (100)	11.82	-	2.97; 24.82	0.007	Increasing
2015–2018	1,064 (38.0)	-	31.18	4.79; 118.80	0.017	Increasing
2018–2023	1,738 (62.0)	-	1.16	27.24; 15.83	0.936	Stable

Data linkage between systems was performed manually, based on rules that included: exact matching of maternal name between syphilis in pregnancy and live birth records; temporal correspondence between maternal diagnosis date and child’s birth date, considering the same interval described above; and exact matching of the mother’s date of birth in both systems.

In cases where discrepancies in names or dates were identified, an individual analysis of spelling similarities and temporal proximity between records was conducted. In the absence of information on gestational trimester, a maximum interval of 10 months between the date of maternal diagnosis and the child’s birth was considered.

The linkage process was subject to limitations inherent to the use of secondary databases, such as inconsistencies in data entry, variations in spelling, and possible typing errors.

The calculations of the syphilis in pregnancy detection rate (Equation A) and the cumulative rate (Equation B) were performed according to the recommendations of the Ministry of Health [6]. 

**Equation A**. Syphilis in pregnancy detection rate


Number of cases of syphilis in pregnant women residing in the municipality in a given year

Total number of live births to women residing in the municipality in the same year
×1000

**Equation B**. Syphilis in pregnancy detection rate


Number of cases of syphilis in pregnant women residing in the municipality during the period

Number of live births to women residing in the municipality at the midpoint of the period (2019)
×1000

### Statistical methods

The detection rate was used as a measure of the frequency of syphilis in pregnancy, calculated by year and for the entire period. The temporal trend analysis of the syphilis in pregnancy detection rate was performed using segmented linear regression, which identifies inflection points indicating significant changes in the trend [19]. 

The Joinpoint Regression Program (version 5.0.2) was used with the following parameters: detection rate as the dependent variable, year of notification as the independent variable, and the Weighted Bayesian Information Criterion as the method for selecting the best-fitting model [19]. Up to two inflection points were automatically tested, heteroscedasticity was assessed using the standard error calculated from the provided data, and the absence of autocorrelation was verified using the Durbin–Watson test (DW 1.890) [19]. 

The mean percent change (MPC) for the entire period and the annual percent changes (APC) for the segments were calculated, with a 95% confidence interval (95%CI) and a significance level of 5%. The trend was classified as increasing (positive APC and p-value<0.050), decreasing (negative APC and p-value<0.050), or stationary (p-value>0.050).

Categorical variables were presented as absolute and relative frequencies. The age variable was described by the median and interquartile range (IQR). Missing data were identified as “not informed” and presented as absolute frequency.

The comparison of the distributions of clinical and epidemiological characteristics among the four periods (P1, P2, P3, and P4) was performed using the Kruskal–Wallis test, with Dunn’s post hoc test, Bonferroni correction, and a significance level of 5%. The Statistical Package for the Social Sciences (SPSS) software (version 25.0) was used for the analyses.

## Results

Between 2015 and 2023, 4,874 cases of syphilis in pregnancy were reported. Among them, 40.8% had no record of non-treponemal titers in SINAN. For this subset, 74.7% of treponemal or non-treponemal test results were located in the IPED/APAE system. After applying the exclusion criteria, the final population comprised 2,802 cases, 79.5% of which were linked to SINASC data, enabling analysis of birth outcomes (Figure 1). 

**Figure 1:**
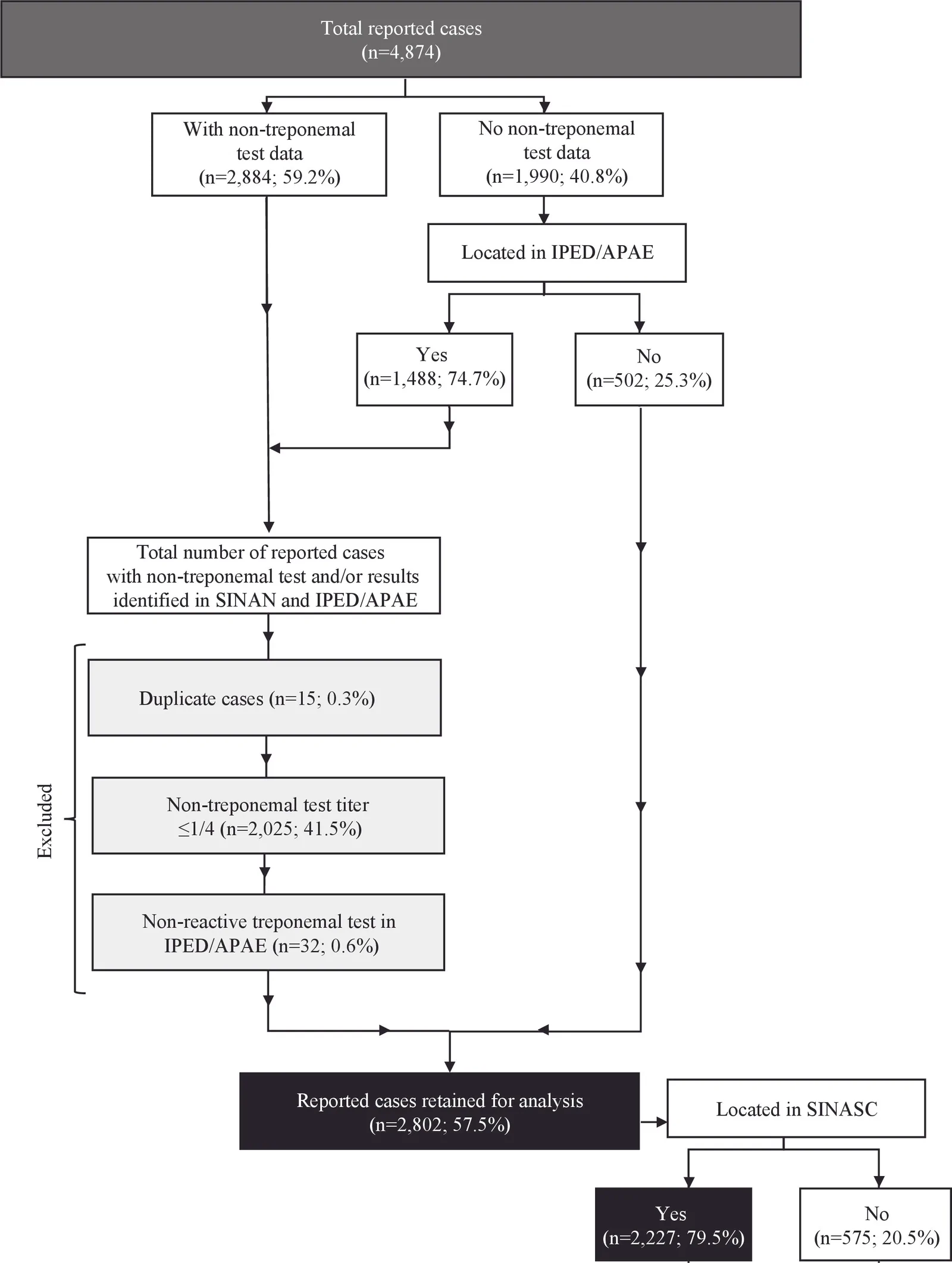
Process of identification and selection of cases of syphilis in pregnancy in the Notifiable Diseases Information System (Sistema de Informação de Agravos de Notificação, SINAN), retrieval of non-treponemal test results from the Institute for Research, Education and Diagnostics of the Association of Parents and Friends of Exceptional Individuals (Instituto de Pesquisas, Ensino e Diagnósticos da Associação de Pais e Amigos dos Excepcionais, IPED/APAE), and identification of births in the Live Birth Information System (Sistema de Informação sobre Nascidos Vivos, SINASC). Campo Grande, 2015–2023 (n=4,874)

During the study period, a marked increase of 170.4% in the syphilis in pregnancy detection rate was observed, with an annual change ranging from 12.5 to 33.8 cases per 1,000 live births (Supplementary Table 2). The temporal trend analysis of the detection rate indicated an increase until 2018 (2015–2018 APC 31.18%; 95%CI 4.79; 118.80; p-value 0.017), followed by stabilization in the subsequent years (2018–2023 APC 1.16%; 95%CI -27.24; 15.83; p-value 0.936) (Table 1). 

**Table 2:** Absolute (n) and relative (%) distribution of sociodemographic characteristics and prenatal care of women reported with syphilis in pregnancy, according to the analyzed periods. Campo Grande, 2015–2023 (n=2,802)

Variables	2015 to 2016-P1^a^		2017 to 2019-P2^b^		2020 to 2021-P3^c^		2022 to 2023-P4^d^		Total	p-value	Comparison between periods
	n (%)		n (%)		n (%)		n (%)		n (%)		Periods (p-value)
**Sociodemographic data**											
**Age group **(years)										0.084	
10–19	125 (31.3)		338 (33.0)		191 (28.2)		192 (27.4)		846 (30.2)		-
20–29	224 (56.1)		538 (52.5)		372 (54.6)		422 (60.4)		1,556 (55.5)		
30–39	47 (11.8)		136 (13.3)		109 (16.0)		81 (11.6)		373 (13.3)		
≥40	3 (0.8)		12 (1.2)		8 (1.2)		4 (0.6)		27 (1.0)		
**Marital status**										0.168	
Single	272 (86.1)		732 (85.9)		500 (89.9)		438 (87.3)		1,942 (87.2)		-
Married/stable union	38 (12.0)		108 (12.7)		46 (8.3)		54 (10.8)		246 (11.1)		
Separated/divorced	6 (1.9)		12 (1.4)		10 (1.8)		10 (2.0)		38 (1.7)		
Not informed	83		172		124		197		576		
**Race/skin color**										0.026	
White	104 (27.7)		280 (27.8)		222 (34.1)		219 (31.7)		825 (30.3)		P2–P3 (0.037)
Brown (Brazilian mixed race)/Black/Asian/Indigenous	271 (72.3)		728 (72.2)		429 (65.9)		472 (68.3)		1,900 (69.7)		
Not informed	24		16		29		8		77		
**Educational level **(**years of schooling**)										0.011	
≤9	166 (53.2)		405 (46.8)		228 (43.6)		243 (41.6)		1,042 (45.6)		P1–P3 (0.023) P1–P4 (0.014)
10–12	134 (42.9)		414 (47.9)		261 (49.9)		320 (54.8)		1,129 (49.4)		
≥13	12 (3.8)		46 (5.3)		34 (6.5)		21 (3.6)		113 (4.9)		
Not informed	87		159		157		115		518		P2–P3 (0.037)
Occupation										0.030	
No	246 (79.1)		607 (72.2)		356 (70.9)		411 (75.8)		1,620 (73.8)		-
Yes	65 (20.9)		234 (27.8)		146 (29.1)		131 (24.2)		576 (26.2)		
Not informed	88		183		178		157		606		
**Prenatal data**											
**Start of prenatal care**										0.164	
Early	241 (79.8)		673 (81.0)		458 (84.8)		396 (80.3)		1,768 (81.6)		-
Late	61 (20.2)		158 (19.0)		82 (15.2)		97 (19.7)		398 (18.4)		
Not informed	97		193		140		206		636		
**Prenatal care visits**										0.005	
None	7 (2.2)		21 (2.5)		14 (2.5)		9 (1.8)		51 (2.3)		P2–P4 (0.003)
1–5	117 (37.4)		330 (38.8)		200 (36.0)		149 (29.8)		796 (35.9)		
≥6	189 (60.4)		500 (58.8)		341 (61.4)		342 (68.4)		1,372 (61.8)		
Not informed	86		173		125		199		583		

^a^Period of penicillin shortage; ^b^Normalization of penicillin supply and pre-COVID-19 pandemic; ^c^COVID-19 pandemic; ^d^ Post-COVID-19 pandemic. The dash indicates that no comparison between specific periods reached a p-value>0.05 after Bonferroni correction.

Regarding the sociodemographic profile, the median age was 22 years (IQR: 5). There was a predominance of single women (87.2%), of Brown/Black/Asian/Indigenous race/skin color (69.7%), with 10 to 12 years of schooling (49.4%), and not employed (73.8%). The proportions of race/skin color and schooling differed among the analyzed periods, with a reduction in the frequency of Brown/Black/Asian/Indigenous women from the pre-pandemic to the pandemic period (72.2% to 65.9%). There was an increase in the proportion of women with 10 to 12 years of schooling from the shortage period to the pandemic period (42.9% to 49.9%) and to the post-pandemic period (42.9% to 54.8%) (Table 2).

Prenatal care was initiated early in 81.6% of cases, and 61.8% of pregnant women attended 6 or more visits, with this proportion increasing from the pandemic to the post-pandemic period (58.8% to 68.4%). Diagnosis of syphilis in pregnancy occurred predominantly in the first trimester (43.4%); however, an increase in late diagnoses from the pandemic to the post-pandemic period was observed (30.5% to 40.2%). There was also a predominance of the latent clinical stage (63.9%), with an increase in this form over the time series (26% to 65.6%, 70.2%, and 70.7%) (Table 3).

**Table 3:** Absolute (n) and relative (%) distribution of clinical, laboratory, and therapeutic characteristics of women reported with syphilis in pregnancy, and characteristics of sexual partner treatment, according to the analyzed periods. Campo Grande, 2015–2023 (n=2,802)

Variables	2015 to 2016-P1^a^		2017 to 2019-P2^b^		2020 to 2021-P3^c^		2022 to 2023-P4^d^		Total	p-value	Comparison between periods
	n (%)		n (%)		n (%)		n (%)		n (%)		Periods (p-value)
**Clinical data**											
**Gestational trimester**										0.021	
First	143 (40.7)		433 (43.1)		281 (47.3)		245 (41.4)		1,102 (43.4)		P3–P4 (0.015)
Second	101 (28.8)		259 (25.8)		132 (22.2)		109 (18.4)		601 (23.7)		
Third	107 (30.5)		312 (31.1)		181 (30.5)		238 (40.2)		838 (33.0)		
Not informed	48		20		86		107		261		
**Clinical classification**										<0.001	
Primary	72 (26.0)		121 (12.0)		82 (12.7)		73 (10.5)		348 (13.2)		P1–P2 (<0.001) P1–P3 (<0.001) P1–P4 (<0.001)
Secondary	14 (5.1)		34 (3.4)		8 (1.2)		12 (1.7)		68 (2.6)		
Tertiary	119 (43.0)		192 (19.0)		103 (15.9)		118 (17.0)		532 (20.2)		
Latent	72 (26.0)		663 (65.6)		454 (70.2)		491 (70.7)		1,680 (63.9)		
Not informed	122		14		33		5		175		P3–P4 (0.015)
**Laboratory data**											
**Prenatal treponemal test**										<0.001	
Non-reactive	7 (1.8)		6 (0.6)		45 (6.7)		27 (4.1)		85 (3.1)		P1–P2 (0.002) P1–P3 (<0.001) P1–P4 (<0.001)
Reactive	345 (87.8)		986 (96.4)		590 (87.4)		603 (90.5)		2,524 (91.5)		
Not performed	41 (10.4)		31 (3.0)		40 (5.9)		36 (5.4)		148 (5.4)		
Not informed	6		1		5		33		45		
**Prenatal non-treponemal test**										<0.001	
Non-reactive	34 (9.4)		31 (3.7)		37 (6.2)		25 (3.8)		127 (5.2)		P1–P2 (<0.001) P1–P3 (0.021) P2–P3 (0.015) P2–P4 (<0.001) P3–P4 (0.001)
Reactive	245 (68.1)		504 (60.4)		384 (64.1)		521 (79.1)		1,654 (67.4)		
Not performed	81 (22.5)		300 (35.9)		178 (29.7)		113 (17.1)		672 (27.4)		
Not informed	39		189		81		40		349		
**Prenatal non-treponemal test titer**										0.031	
1:8 to 1:32	252 (77.3)		579 (72.0)		401 (70.0)		460 (76.6)		1,692 (73.4)		-
>1:32 and ≤1:64	64 (19.9)		210 (26.3)		163 (28.4)		131 (21.8)		568 (24.7)		
>1:128	9 (2.8)		14 (1.7)		8 (1.6)		10 (1.7)		41 (1.8)		
**Treatment data**											
**Maternal treatment**										<0.001	
With penicillin	359 (91.8)		985 (96.4)		622 (92.6)		623 (91.43)		2589 (93.6)		P2–P3 (0.004) P2–P4 (0.001)
Without penicillin	6 (1.5)		0 (0.0)		1 (0.1)		4 (0.6)		11 (0.4)		
Not performed	26 (6.6)		37 (3.6)		49 (7.3)		55 (8.1)		167 (6.0)		
Not informed	9		2		9		15		35		
**Treatment adequacy^a^**										0.004	
Inadequate	96 (36.4)		149 (15.4)		99 (16.7)		102 (16.5)		446 (18.2)		P1–P2 (0.001) P1–P3 (0.001) P1–P4 (0.001)
Adequate	168 (63.4)		819 (84.6)		494 (83.3)		517 (83.5)		1,998 (81.8)		
Not informed											
**Sexual partner data**											
**Partner treatment**										0.001	
Not performed	143 (39.7)		457 (47.8)		278 (49.8)		265 (53.9)		1,143 (48.3)		P1–P2 (0.017) P1–P3 (0.008) P1–P4 (0.001)
With penicillin	206 (57.2)		496 (51.8)		274 (49.1)		215 (43.7)		1,191 (50.3)		
Without penicillin	11 (3.1)		4 (0.4)		6 (1.1)		12 (2.4)		33 (1.4)		
Not informed	39		67		122		207		435		
**Reason for no treatment**										0.001	
Had no further contact	72 (46.5)		247 (48.5)		102 (34.3)		89 (25.0)		510 (38.7)		P1–P4 (0.001) P2–P3 (0.001) P2–P4 (0.001) P3–P4 (0.001)
Not communicated/summoned the health unit	2 (1.3)		31 (6.1)		11 (3.7)		8 (2.2)		52 (3.9)		
Did not attend for treatment	23 (14.8)		107 (21.0)		56 (18.9)		38 (10.7)		224 (17.0)		
Refused treatment	14 (9.0)		11 (2.2)		8 (2.7)		8 (2.2)		41 (3.1)		
Non-reactive serology	9 (5.8)		34 (6.7)		18 (6.1)		15 (4.2)		76 (5.8)		
Another reason	35 (22.6)		79 (15.5)		102 (34.3)		198 (55.6)		414 (1.4)		

^a^Period of penicillin shortage; ^b^Normalization of penicillin supply and pre-COVID-19 pandemic; ^c^COVID-19 pandemic; ^d^ Post-COVID-19 pandemic. The dash indicates that no comparisons between specific periods reached a p-value0.05 after Bonferroni correction.

Most treponemal (91.5%) and non-treponemal (67.4%) tests performed during prenatal care were reactive, with the proportion of reactive results increasing over the time series. A higher frequency of non-treponemal titers between 1:8 and 1:32 (73.4%) was observed, with no differences among the analyzed periods (Table 3).

Benzathine penicillin G was prescribed to 93.6% of women, and the dose was considered adequate in 81.8% of cases. A reduction in penicillin prescription (96.4% to 92.6% and 91.4%) and an increase in non-performance of treatment from the pre-pandemic to the pandemic and post-pandemic periods (3.6% to 7.3% and 8.1%) were observed. On the other hand, treatment adequacy increased after the penicillin shortage period (63.4% to 84.6%, 83.3%, and 83.5%) (Table 3).

Among sexual partners, treatment was not prescribed in 48.3% of cases, with a progressive increase in the absence of treatment from the pre-pandemic period onward (39.7% to 47.8%, 49.8%, and 53.9%). The main reasons for non-prescription were lack of contact with the woman (38.7%) and other reasons (31.4%), with differences in proportions across the analyzed periods (p-value0.001) (Table 3).

Among the matched births, there was a predominance of male newborns (51.3%), born at term (87.2%), with adequate birth weight (84.4%), without indication of asphyxia at 1 (95.4%) and 5 (98.9%) minutes, and without congenital anomalies (99.2%). None of these variables showed differences over time (Table 4).

In total, 18 newborns (0.8%) presented congenital anomalies, of whom 33.3% had more than one anomaly. A total of 21 distinct anomalies were identified, of which 9 (42.9%) corresponded to musculoskeletal malformations and deformities; 4 (19.0%) to genital organ anomalies; 3 (14.3%) to anomalies of the eye, ear, face, and neck; 2 (9.5%) to circulatory system anomalies; 1 (4.8%) to cleft lip and cleft palate; one (4.8%) to nervous system anomalies; and 1 (4.8%) to respiratory system anomalies. Due to the low frequency, these data were not tabulated.

## Discussion

In this study, the detection rate of syphilis in pregnancy increased markedly in Campo Grande, with a rising trend until 2018 and stabilization in the subsequent years. Despite advances in public policies aimed at strengthening prenatal care, expanding testing, and standardizing the clinical management of syphilis in pregnancy in Brazil [20-21], the analysis of the historical series from a capital city in the Central-West region revealed that weaknesses in timely diagnosis and, especially, in partner treatment—key aspects for disease control—persist. The differences observed between periods suggest changes in the healthcare profile of syphilis in pregnancy, with improvement in maternal treatment in the pre-pandemic period, changes in the sociodemographic profile during the pandemic, and an increase in late diagnoses in the post-pandemic period.

This study had limitations inherent in the use of secondary data, including underreporting, data-entry errors, and compromised information quality due to missing fields. It was observed that approximately 20% of cases of syphilis in pregnancy were not linked to SINASC, which may reflect unrecorded fetal losses or underreporting of adverse outcomes. These findings indicate potential information biases and reinforce the need to improve data-entry quality and information-system integration. 

Manual matching between databases may have introduced biases due to classification errors, selective record losses, and variations in information quality over time, which may have partially compromised the comparison of findings. Finally, the annual aggregation of data may have introduced temporal bias by failing to capture intra-annual variations and seasonal fluctuations, thereby limiting the general characterization of the results and the precision of the estimates.

The temporal trend observed in Campo Grande showed a pattern distinct from that described in Brazil, where a continuous increasing trend in the detection rate of syphilis in pregnancy has been observed from [6,22] 2014 to 2023 (APC 11.15; 95%CI 2.78; 23.19), as well as in the five regions of the country [22]. It is noteworthy that these studies did not exclude possible cases of serological scar, which may have influenced the estimates. 

The stabilization observed from 2018 onward in this analysis may be associated with a set of factors, including improvements in surveillance services [23], expansion of early diagnosis, persistence of gaps in prenatal follow-up and adequate treatment of pregnant women and their partners [3,4], as well as events with greater systemic impact, such as the penicillin shortage [7,24] and restrictions on access to health services during the COVID-19 pandemic [10-14]. Thus, the stabilization of the detection rate of syphilis in pregnancy should be interpreted with caution, as it may reflect both advances in surveillance and care as well as limitations of services that hindered timely detection and favored underreporting.

The higher frequency of the disease among Brown/Black/Asian/Indigenous women, young adults, and those not employed was similar to the pattern identified in Mato Grosso do Sul from 2008 to 2018 [25] and in Brazil in 2024 [6,26], where cases were also concentrated in these groups. This profile is consistent with national sociodemographic inequalities, which are frequently associated with barriers to timely access to health services [25,27].

In Brazil, regarding the 2011–2018 data analysis, compared with White women, Brown and Black women have a higher probability of inadequate treatment (adjusted OR_Brown_ 1.10; 95%CI 1.01; 1.24; adjusted OR_Black_ 1.23; 95%CI 1.07; 1.41), late diagnosis (adjusted OR_Brown_ 1.13; 95%CI 1.08; 1.17; adjusted OR_Black_ 1.17; 95%CI 1.11; 1.24), and a lower probability of partner treatment (adjusted OR_Brown_ 1.11; 95%CI 1.05; 1.17; adjusted OR_Black_ 1.11; 95%CI 1.03; 1.20) [27]. The educational level should be analyzed both in the context of the overall increase in educational attainment of the Brazilian population (2016–2024) [28] and the spread of syphilis in pregnancy across diverse socioeconomic settings and levels of development [1,5], extending beyond traditionally more vulnerable groups [6,26]. 

Across the analyzed series, the disease characteristics showed significant changes between periods. From 2014 onward, reduced penicillin production, together with other logistical issues, led to a global shortage [7,8], which coincided with a 92.1% increase in the detection rate of syphilis in pregnancy in 2017 [6] and a greater impairment of maternal treatment [29]. In an attempt to mitigate the shortage of the medication, its use was prioritized for the treatment of pregnant women and children exposed to syphilis [8]. This effort, together with the stabilization process, likely contributed to the reduction in the prescribing of other medications and the increase in the frequency of adequate treatment during 2017–2019.

Even with the availability of medication, the high frequency of untreated partners increases the likelihood of reinfection among pregnant people, since adequate treatment, combined with partner treatment, represents a reduction in the risk of congenital syphilis (adjusted RR 0.60; 95%CI 0.55; 0.66) [4].

From 2017 to 2019, the “Syphilis No” Project (*Projeto Sífilis Não*) was implemented in Campo Grande. This was a national strategy that promoted the strengthening and expansion of surveillance, diagnosis, and treatment actions for syphilis through the qualification of health teams, structuring of services, expansion of testing, regularization of penicillin supply, and reduction of vertical transmission [30]. However, the findings of this study indicated that, even with the expansion of the response capacity of services, failures in screening for syphilis in pregnancy persisted, associated with gaps in prenatal follow-up, partner management, and limitations in the functioning of services, which may have restricted the impact of the implemented actions. 

Between 2020 and 2021, the COVID-19 pandemic compromised the effectiveness of syphilis control actions due to the partial interruption of health service operations [10-12]. In Brazil, a reduction of approximately 2.65 million diagnostic and therapeutic procedures related to syphilis was estimated in 2020 compared with the average for the period 2016–2019, with a more pronounced decline in Mato Grosso do Sul (63%) [12]. This context may have contributed to the reduced performance of treponemal and non-treponemal tests and to the maintenance of the high titers observed in this study, consistent with delays in diagnosis and initiation of treatment.

In this study, a 29% reduction in the detection rate of syphilis in pregnancy was observed between 2020 and 2021, in contrast to the 16% increase observed nationally in the same period [6]. The redirection of surveillance actions toward pandemic response, combined with structural challenges in health services, may have contributed to overall underreporting of cases [31], indicating that the observed decrease likely reflects limitations in detection and reporting rather than a real reduction in disease occurrence.

The pandemic and measures restricting social contact intensified structural and health inequalities worldwide [32]. The reduction observed in this study in cases among Black/Brown/Asian/Indigenous women during this period may be related to increased barriers to access health services and case detection in a structurally unequal system [25]. This highlights the need for targeted strategies to reduce inequities in access, especially during public health emergencies. 

In this study, during the post-pandemic period (2021–2022), the frequency of prenatal care visits was higher compared with the pandemic period. This reflects the resumption of health service operations and the reestablishment of routine care. This recovery may have contributed to stabilizing the trend in the detection rate observed in this study, since adequate prenatal follow-up favors timely screening, diagnosis, and treatment of syphilis, reducing the risk of infection during pregnancy [3] and adverse neonatal outcomes [2-4,9]. 

Despite the increase in prenatal visits and early initiation of prenatal care throughout the series analyzed in this study, the predominance of late diagnoses (56%) and advanced clinical stages (64%) of syphilis suggests the persistence of failures in maternal diagnosis and treatment. Delayed diagnosis compromises the timely initiation of treatment, increasing the risk of vertical transmission and adverse neonatal outcomes [2-4]. The number of prenatal care visits alone is not sufficient to prevent vertical transmission when opportunities for testing and treatment are missed.

Despite its limitations, in the investigated context, this study enabled monitoring of changes in detection trends and analysis of variations in vulnerability and risk profiles over time, providing relevant evidence to guide public policies. The use of SINAN and SINASC allowed the expansion of clinical information and improved data quality, which represents an important strength of the study. 

The detailed analysis of the clinical and epidemiological characteristics of syphilis in pregnancy in periods marked by events of public health relevance provides an integrated view of changes in the magnitude of the disease and in the profile of the affected population. The findings reinforced the need for continuous strengthening of epidemiological surveillance, with effective integration between information systems, improvement of prenatal care, and active monitoring of treatment among sexual partners, especially in scenarios of public health crises.

Syphilis in pregnancy showed an increase in the detection rate until 2018, followed by stabilization, despite advances in maternal diagnosis and treatment. The persistence of failures in partner treatment and variations in healthcare delivery highlight the importance of using epidemiological evidence to improve health management and strengthen public policies for prevention and control.

## Data Availability

The anonymized dataset and analysis script used in this study may be made available from the authors upon a reasonable and justified request following publication.
