# Supplementary material for: Syphilis in pregnancy detection rate: time series study associated with descriptive analysis by periods, Campo Grande, 2015–2023
Source: Epidemiol Serv Saude. 2026 Jul 31;35:e20250940. doi: 10.1590/S2237-96222026v35e20250940.en (PMC13686406; doi:10.1590/S2237-96222026v35e20250940.en)
Supplement: Translation

# Translation: Taxa de detecção de sífilis gestacional: estudo de série temporal associado à análise descritiva por períodos, Campo Grande, 2015-2023

**DOI:** 10.1590/S2237-96222026v35e20250940.pt

**Authors:** Caroliny Oviedo Fernandes Reis, Luciana Aparecida da Cunha Borges, Cláudia Du Bocage Santos-Pinto, Ana Lúcia Lyrio de Oliveira, Daniele Soares-Marangoni, Carlos Dornels Souza Freire, Everton Falcão de Oliveira

**Affiliations:** 1 Universidade Federal de Mato Grosso do Sul Programa de Pós-Graduação em Doenças Infecciosas e Parasitárias Campo Grande MS Brasil Universidade Federal de Mato Grosso do Sul, Programa de Pós-Graduação em Doenças Infecciosas e Parasitárias, Campo Grande, MS, Brasil; 2 Universidade Federal de Mato Grosso do Sul Faculdade de Medicina Campo Grande MS Brasil Universidade Federal de Mato Grosso do Sul, Faculdade de Medicina, Campo Grande, MS, Brasil; 3 Universidade Federal de Mato Grosso do Sul Instituto Integrado de Saúde Campo Grande MS Brasil Universidade Federal de Mato Grosso do Sul, Instituto Integrado de Saúde, Campo Grande, MS, Brasil; 4 Universidade Federal do Vale do São Francisco Faculdade de Medicina Petrolina PE Brasil Universidade Federal do Vale do São Francisco, Faculdade de Medicina, Petrolina, PE, Brasil

**Keywords:** Sífilis, Gravidez, Notificação de Doenças, Sistemas de Informação em Saúde, Estudos de Séries Temporais

## Abstract

**Objetivo:**

Analisar série temporal e tendência da taxa de detecção de sífilis gestacional em nove anos e variações das características clínico-epidemiológicas em quatro períodos segundo eventos sanitários relevantes em saúde pública em Campo Grande, Mato Grosso do Sul.

**Métodos:**

Estudo de série temporal com análise descritiva por períodos, utilizando dados dos sistemas de Informação de Agravos de Notificação e de Informações sobre Nascidos Vivos. Utilizaram-se regressão linear segmentada, para analisar a tendência temporal da taxa de detecção de sífilis gestacional, e teste Kruskal-Wallis, para analisar as variáveis clínico-epidemiológicas entre os períodos avaliados: desabastecimento de penicilina (2015-2016); normalização do abastecimento e período de pré-pandemia (2017-2019); pandemia (2020-2021); e pós-pandemia de covid-19 (2022-2023).

**Resultados:**

Analisaram-se 2.802 casos. Observaram-se aumento de 170,4% na taxa de detecção, com tendência crescente até 2018 (variação percentual anual, VPA 31,18%; intervalo de confiança de 95%, IC95% 4,79; 118,80; p-valor 0,017) e estabilização posterior (VPA 1,16%; IC95% -27,24; 15,83; p-valor 0,936). Nas características clínico-epidemiológicas, verificaram-se aumento da frequência da fase latente, da positividade de testes treponêmicos, do tratamento adequado e da escolaridade da mulher, e redução do tratamento dos parceiros. No período 2017-2019, houve redução no uso de testes não treponêmicos; 2020-2021, redução de mulheres pardas/pretas/amarelas/indígenas; e 2022-2023, aumento de consultas de pré-natal e diagnósticos tardios.

**Conclusão:**

A taxa de detecção de sífilis gestacional aumentou até 2018 e permaneceu estável posteriormente. As mudanças nas características clínico-epidemiológicas refletem variações no contexto assistencial, com avanços no cuidado pré-natal, mas persistência de fragilidades no diagnóstico oportuno e no tratamento dos parceiros.

**Aspectos éticos**

Esta pesquisa respeitou os princípios éticos, obtendo os seguintes dados de aprovação:

Comitê de ética em pesquisa Universidade Federal de Mato Grosso do Sul

Número do parecer 5.080.108

Data de aprovação 4/11/2021

Certificado de apresentação de apreciação ética 52447421.0.0000.0021

Registro do consentimento livre e esclarecido Dispensado.

## Introdução

A sífilis, uma infecção sexualmente transmissível causada pelo *Treponema pallidum*, é considerada um problema de saúde pública global, com aumento contínuo de casos desde os anos 2000, apesar de tratamento efetivo estabelecido [1]. A sífilis transmitida verticalmente contribui para que haja abortos, natimortos e óbitos neonatais, além de aumentar o risco de baixo peso ao nascer, a prematuridade e a possibilidade de danos irreversíveis como surdez, alterações ósseas e neurológicas [1-5]. 

Em 2016, estimaram-se 988 mil prováveis casos de sífilis ativa em gestantes no mundo todo, com maior carga no continente africano e frequência relevante nas Américas. Destes, 661 mil foram casos de sífilis congênita, dos quais 54% apresentaram eventos adversos, incluindo óbitos fetais e natimortos (40%), manifestações clínicas da sífilis congênita (31%), morte neonatal (17%) e prematuridade ou baixo peso ao nascer (12%) [1]. No Brasil, de 2013 a 2024, observou-se aumento de 295% da taxa de detecção de sífilis gestacional, 74% na incidência de sífilis congênita e 33% na mortalidade infantil específica por sífilis congênita [6].

Houve eventos relevantes em saúde pública que podem ter comprometido estratégias de controle e prevenção da sífilis. Um exemplo foi a escassez mundial de penicilina entre 2014 e 2016 [7,8]. Isso impactou negativamente o tratamento de sífilis em gestantes, um dos pilares na prevenção da transmissão vertical [1,9]. Entre 2014 e 2018, o Brasil registrou aumento de 65% da taxa de incidência de sífilis congênita e de 43% da taxa de óbitos por sífilis em menores de 1 ano de idade [6]. 

Outro evento de relevância sanitária foi a pandemia de covid-19 [10,11]. Medidas como isolamento social e redução na oferta de serviços essenciais, como transporte público e saúde, diminuíram a busca pelos serviços de saúde sexual e reprodutiva, com impacto direto no diagnóstico oportuno e tratamento [10-13]. Após a pandemia, os serviços enfrentaram acúmulo de demandas e agravamento de condições evitáveis devido à desassistência prolongada [14].

Com o crescente aumento dos casos de sífilis em gestantes, o estudo da tendência temporal torna-se fundamental para descrever a evolução da doença ao longo do tempo e identificar períodos com possíveis fragilidades na resposta dos serviços de saúde. Entretanto, ainda existem incertezas sobre como eventos de relevância em saúde pública, como o desabastecimento de penicilina e a pandemia de covid-19, se associaram às mudanças nas características clínico-epidemiológicas da sífilis gestacional. 

Este estudo objetivou analisar a série temporal e a tendência da taxa de detecção de sífilis gestacional ao longo de nove anos. Também se propôs analisar as frequências das características clínico-epidemiológicas da doença em quatro períodos definidos segundo eventos sanitários relevantes em saúde pública em Campo Grande, Mato Grosso do Sul.

## Métodos

### Delineamento

Trata-se de estudo de série temporal com análise descritiva por períodos, que observou o comportamento da taxa de detecção da sífilis gestacional e as variações clínico-epidemiológicas em quatro períodos de 2015 a 2023, em Campo Grande, Mato Grosso do Sul. Os períodos foram definidos de acordo com a ocorrência de eventos de importância em saúde pública, apontados como possíveis fatores de agravamento de algumas condições de saúde, incluindo a sífilis.

- Período P1 (2015-2016): desabastecimento mundial de penicilina, com 60,7% dos estados brasileiros relatando escassez [15].
- Período P2 (2017-2019): normalização progressiva do abastecimento no Brasil, antes da pandemia de covid-19 [7,8,11].
- Período P3 (2020-2021): pandemia de covid-19, com interrupções da oferta assistencial de rotina nos serviços de saúde [11].
- Período P4 (2022-2023): pós-pandemia com a retomada gradual da oferta assistencial dos serviços e programas de rotina [14].

### Contexto

Campo Grande, capital de Mato Grosso do Sul, está localizada na região Centro-Oeste do Brasil e, em 2022, tinha 898.100 habitantes [16]. Destes, 271.901 eram mulheres em idade fértil [16]. No município, foi registrada média de 13.412,8 nascidos vivos entre 2015 e 2023 [17]. 

### Participantes

Foram considerados elegíveis todos os casos de sífilis gestacional reportados ao Sistema de Informação de Agravos de Notificação (Sinan) no período 2015-2023. Excluíram-se os casos duplicados e os resultados de titulação de teste não treponêmico≤1:4, pois, conforme diretrizes do Ministério da Saúde, são indicativos de cicatriz sorológica na ausência de evidência clínica ou epidemiológica de infecção ativa [9]. Também foram utilizados dados de nascimento da criança gestada do Sistema de Informações sobre Nascidos Vivos (Sinasc).

### Variáveis

As seguintes variáveis foram extraídas do Sinan: idade (em anos contínuo), faixa etária (10-19, 20-29, 30-39, ≥40 anos), raça/cor da pele (branca, parda/preta/amarela/ indígena, não informado), escolaridade (≤9 anos, 10-12 anos, ≥13 anos, não informado), ocupação (sim, não, não informado), idade gestacional no diagnóstico (primeiro, segundo, terceiro, não informado), classificação clínica da sífilis (primária, secundária, terciária, latente, não informado), testes treponêmico (reagente, não reagente, não realizado, não informado) e não treponêmico (reagente, não reagente, não realizado, não informado) no pré-natal, titulação não treponêmica (1:8-1:32, <1:32 e ≤1:64, ≥1:128), tratamento prescrito à mulher (com penicilina, sem penicilina, não realizado, não informado), adequação do tratamento materno (adequado, inadequado, não informado), tratamento do parceiro (não realizado, com penicilina, sem penicilina, não informado) e motivo de não tratamento do parceiro (não teve mais contato, não comunicado ou convocado à unidade de saúde, não compareceu para tratamento, recusou tratamento, sorologia não reagente, outro motivo). 

A variável raça/cor da pele é autorreferida de forma individual no momento da notificação, porém, para este estudo, foi agrupada em três categorias, conforme descrito anteriormente. Para a variável “adequação do tratamento”, considerou-se a dose preconizada pelo Ministério da Saúde para cada fase clínica da sífilis conforme o ano [9,18]. 

Do Sinasc foram utilizadas as variáveis: estado civil (solteira, casada ou união estável, separada ou divorciada, não informado), número de consultas (nenhuma, 1-5, 6 ou mais, não informado) e início do pré-natal (precoce, tardio, não informado), Apgar no primeiro e quinto minutos (sem asfixia, asfixia moderada, asfixia grave, não informado), idade gestacional (prematuro, a termo, pós-termo, não informado), peso ao nascer (baixo peso, peso normal, macrossômico, não informado) e presença de anomalias congênitas (não, sim, não informado).

### **Fonte de dados**/mensuração

Dados nominais do Sinan e do Sinasc foram fornecidos pela Secretaria Municipal de Saúde de Campo Grande. Na ausência de titulações não treponêmicas nas notificações do Sinan, realizou-se a busca desses resultados no sistema eletrônico do Instituto de Pesquisas, Ensino e Diagnósticos da Associação de Pais e Amigos dos Excepcionais de Campo Grande (Iped/Apae). Trata-se de laboratório de análises clínicas responsável pela triagem pré-natal das gestantes atendidas pelo Sistema Único de Saúde e instituições parceiras em Mato Grosso do Sul. 

A busca se deu fornecendo-se nome, data de nascimento e nome da mãe da mulher. Considerou-se um intervalo retrospectivo máximo definido de acordo com o trimestre gestacional registrado no Sinan, contado a partir da data da notificação: até 10 meses para o primeiro trimestre, até 7 meses para o segundo e até 4 meses para o terceiro trimestre. Esses intervalos contemplaram o período esperado de realização dos exames laboratoriais de rotina no pré-natal, conforme a idade gestacional no momento da notificação.

Em seguida, foram identificados e excluídos registros duplicados. Estes foram definidos como notificações repetidas de sífilis gestacional do mesmo episódio da doença para a mesma mulher. Na identificação de duplicidades, consideraram-se a data do diagnóstico, o trimestre gestacional informado e a titulação não treponêmica (Tabela Suplementar 1). 

A vinculação de dados entre os sistemas foi realizada manualmente, com base em regras que incluíram: compatibilidade exata do nome materno entre os registros de sífilis gestacional e de nascido vivo; correspondência temporal entre a data do diagnóstico materno e a data de nascimento da criança, considerando o mesmo intervalo descrito anteriormente; e compatibilidade exata da data de nascimento da mãe em ambos os sistemas.

Nos casos em que houve discrepâncias em relação a nomes ou datas, realizou-se análise individual das similaridades de grafia e da proximidade temporal entre os registros. Na ausência de informação sobre o trimestre gestacional, considerou-se o intervalo máximo de 10 meses entre a data do diagnóstico materno e o nascimento da criança.

A vinculação esteve sujeita a limitações do uso de bases secundárias, como inconsistências de preenchimento, variações na grafia e possíveis erros de digitação.

Os cálculos das taxas de detecção da sífilis gestacional (Equação A) e da taxa cumulativa (Equação B) foram realizados de acordo com as recomendações do Ministério da Saúde [6]. 

**Equação A**. Taxa de detecção de sífilis gestacional

$$\frac{\text{ Nº de casos de sífilis em gestantes residentes no município no ano }}{\text{ Total de nascidos vivos de mulheres residentes no município no ano }}\times 1000$$

**Equação B**. Taxa de detecção de sífilis gestacional

$$\frac{\text{ Nº de casos de sífilis em gestantes residentes no município no período }}{\text{ Nº de nascidos vivos de mulheres residentes no município na metade do período (2019) }}\times 1000$$

### Métodos estatísticos

A taxa de detecção foi utilizada como medida de frequência da sífilis gestacional, calculada por ano e para o período. A análise de tendência temporal da taxa de detecção de sífilis gestacional foi realizada por meio de regressão linear segmentada que identifica pontos de inflexão em mudanças significativas nas tendências temporais [19]. 

O software Joinpoint Regression Program (versão 5.0.2) foi utilizado e com os seguintes parâmetros: a taxa de detecção como variável dependente, o ano de notificação como variável independente e critério de informação bayesiano ponderado como método de seleção do modelo com melhor ajuste [19]. Até dois pontos de inflexão foram testados automaticamente, com heterocedasticidade determinada pelo erro-padrão calculado com base nos dados fornecidos e ausência de autocorrelação verificada pelo teste de Durbin-Watson (DW 1,890) [19]. 

Foram calculadas a variação percentual média (VPM) para o período total e as variações percentuais anuais (VPA) para os seguimentos, com intervalo de confiança de 95% (IC95%) e nível de significância de 5%. A tendência foi classificada como crescente (VPA positiva e p-valor<0,050), decrescente (VPA negativa e p-valor<0,050) ou estacionária (p-valor>0,050).

As variáveis categóricas foram apresentadas por meio das frequências absoluta e relativa. A variável idade foi descrita pela mediana e intervalo interquartil (IIQ). Os dados ausentes foram identificados como “não informados” e apresentados em frequência absoluta.

A comparação das distribuições das características clínico-epidemiológicas entre os quatro períodos (P1, P2, P3 e P4) foi realizada por meio do teste de Kruskal-Wallis, com pós-teste de Dunn, correção de Bonferroni e nível de significância de 5%. Utilizou-se o software Statistical Package for the Social Sciences (versão 25.0) para as análises.

## Resultados

Entre 2015 e 2023, foram notificados 4.874 casos de sífilis gestacional. Entre eles, 40,8% não apresentavam registro da titulação não treponêmica no Sinan. Para esse subconjunto, 74,7% dos resultados de testes treponêmicos ou não treponêmicos foram localizados no sistema do Iped/Apae. Após aplicação de critérios de exclusão, a população final foi composta por 2.802 casos, dos quais 79,5% apresentaram vinculação com dados no Sinasc, o que possibilitou a análise dos desfechos ao nascimento (Figura 1). 

**Figura 1:** Processo de identificação e seleção dos casos de sífilis gestacional no Sistema de Informação de Agravos de Notificação (Sinan), localização de resultados de testes não treponêmicos no Pesquisas, Ensino e Diagnósticos da Associação de Pais e Amigos dos Excepcionais (Iped/Apae) e identificação de nascimentos no Sistema de Informações sobre Nascidos Vivos. Campo Grande, 2015-2023 (n=4.874)

No período em análise, observou-se aumento expressivo da taxa de detecção de sífilis gestacional em 170,4%, com variação anual de 12,5 a 33,8 casos/1 mil nascidos vivos (Tabela Suplementar 2). A análise de tendência temporal da taxa de detecção indicou crescimento até 2018 (2015-2018: VPA 31,18%; IC95% 4,79; 118,80; p-valor 0,017), seguido de estabilização nos anos seguintes (2018-2023: VPA 1,16%; IC95% -27,24; 15,83; p-valor 0,936) (Tabela 1). 

**Tabela 1:** Tendência temporal da taxa de detecção de sífilis gestacional por 1 mil nascidos vivos, variação percentual anual (VPA), variação percentual média (VPM) e intervalo de confiança de 95% (IC95%). Campo Grande, 2015-2023 (n=2.802)

Período	n (%)	VPM	VPA	IC95%	p-valor	Tendência
2015-2023	2.802 (100)	11,82	-	2,97; 24,82	0,007	Crescente
2015-2018	1.064 (38,0)	-	31,18	4,79; 118,80	0,017	Crescente
2018-2023	1.738 (62,0)	-	1,16	27,24; 15,83	0,936	Estacionária

No perfil sociodemográfico, a idade mediana foi de 22 anos (IIQ:5). Observou-se predominância de mulheres solteiras (87,2%), de raça/cor da pele parda/preta/indígena/amarela (69,7%), com escolaridade entre 10 e 12 anos (49,4%) e sem ocupação (73,8%). As proporções das variáveis raça/cor da pele e escolaridade diferiram entre os períodos analisados, com redução da frequência de mulheres pretas/pardas/indígenas/amarelas do período pré-pandêmico para o pandêmico (72,2% para 65,9%). Ocorreu aumento da proporção de mulheres com 10 a 12 anos de estudo do período de desabastecimento para os períodos pandêmico (42,9% para 49,9%) e pós-pandêmico (42,9% para 54,8%) (Tabela 2).

**Tabela 2:** Distribuições absoluta (n) e relativa (%) das características sociodemográficas e do pré-natal das mulheres notificadas com sífilis gestacional segundo os períodos analisados. Campo Grande, 2015-2023 (n=2.802)

Variáveis	2015 a 2016-P1^a^		2017 a 2019-P2^b^		2020 a 2021-P3^c^		2022 a 2023-P4^d^		Total	p-valor	Comparação entre período
	n (%)		n (%)		n (%)		n (%)		n (%)		Períodos (p-valor)
**Dados sociodemográficos**											
**Faixa etária **(anos)										0,084	
10-19	125 (31,3)		338 (33,0)		191 (28,2		192 (27,4)		846 (30,2)		-
20-29	224 (56,1)		538 (52,5)		372 (54,6)		422 (60,4)		1.556 (55,5)		
30-39	47 (11,8)		136 (13,3)		109 (16,0)		81 (11,6)		373 (13,3)		
≥40	3 (0,8)		12 (1,2)		8 (1,2)		4 (0,6)		27 (1,0)		
**Estado civil**										0,168	
Solteira	272 (86,1)		732 (85,9)		500 (89,9)		438 (87,3)		1.942 (87,2)		-
Casada/união estável	38 (12,0)		108 (12,7)		46 (8,3)		54 (10,8)		246 (11,1)		
Separada/divorciada	6 (1,9)		12 (1,4)		10 (1,8)		10 (2,0)		38 (1,7)		
Não informado	83		172		124		197		576		
**Raça/cor da pele**										0,026	
Branca	104 (27,7)		280 (27,8)		222 (34,1)		219 (31,7)		825 (30,3)		P2-P3 (0,037)
Pardas/pretas/indígenas/amarelas	271 (72,3)		728 (72,2)		429 (65,9)		472 (68,3)		1.900 (69,7)		
Não informado	24		16		29		8		77		
**Escolaridade **(anos)										0,011	
≤9	166 (53,2)		405 (46,8)		228 (43,6)		243 (41,6)		1.042 (45,6)		P1-P3 (0,023) P1-P4 (0,014)
10-12	134 (42,9)		414 (47,9)		261 (49,9)		320 (54,8)		1.129 (49,4)		
≥13	12 (3,8)		46 (5,3)		34 (6,5)		21 (3,6)		113 (4,9)		
Não informado	87		159		157		115		518		
Ocupação										0,030	
Não	246 (79,1)		607 (72,2)		356 (70,9)		411 (75,8)		1.620 (73,8)		-
Sim	65 (20,9)		234 (27,8)		146 (29,1)		131 (24,2)		576 (26,2)		
Não informado	88		183		178		157		606		
**Dados de pré**-natal											
**Início de pré**-natal										0,164	
Precoce	241 (79,8)		673 (81,0)		458 (84,8)		396 (80,3)		1.768 (81,6)		-
Tardio	61 (20,2)		158 (19,0)		82 (15,2)		97 (19,7)		398 (18,4)		
Não informado	97		193		140		206		636		
**Consultas de pré**-natal										0,005	
Nenhuma	7 (2,2)		21 (2,5)		14 (2,5)		9 (1,8)		51 (2,3)		P2-P4 (0,003)
1-5	117 (37,4)		330 (38,8)		200 (36,0)		149 (29,8)		796 (35,9)		
≥6	189 (60,4)		500 (58,8)		341 (61,4)		342 (68,4)		1.372 (61,8)		
Não informado	86		173		125		199		583		

^a^Período de desabastecimento de penicilina; ^b^Normalização da produção de penicilina e pré-pandemia de covid-19; ^c^Período de pandemia de covid-19; ^d^Período pós-pandemia de covid-19. O traço indica que nenhuma comparação entre períodos específicos atingiu p-valor>0,005 após a correção com Bonferroni.

O pré-natal foi iniciado precocemente em 81,6% dos casos, e 61,8% das gestantes realizaram 6 ou mais consultas, observando-se aumento dessa proporção do período pandêmico para o pós-pandêmico (58,8% para 68,4%). O diagnóstico de sífilis gestacional ocorreu predominantemente no primeiro trimestre (43,4%); entretanto, verificou-se aumento de diagnósticos tardios do período pandêmico para o pós-pandêmico (30,5% para 40,2%). Houve ainda predomínio da fase clínica latente (63,9%), com aumento dessa forma ao longo da série temporal (26,0% para 65,6%, 70,2% e 70,7%) (Tabela 3).

**Tabela 3:** Distribuições absoluta (n) e relativa (%) das características clínicas, laboratoriais e terapêuticas das mulheres notificadas com sífilis gestacional e características do tratamento da parceria sexual segundo os períodos analisados. Campo Grande, 2015-2023 (n=2.802)

Variáveis	2015 a 2016-P1^a^		2017 a 2019-P2^b^		2020 a 2021-P3^c^		2022 a 2023-P4^d^		Total	p-valor	Comparação entre período
	n (%)		n (%)		n (%)		n (%)		n (%)		Períodos (p-valor)
**Dados clínicos**											
**Trimestre gestacional**										0,021	
Primeiro	143 (40,7)		433 (43,1)		281 (47,3)		245 (41,4)		1.102 (43,4)		P3-P4 (0,015)
Segundo	101 (28,8)		259 (25,8)		132 (22,2)		109 (18,4)		601 (23,7)		
Terceiro	107 (30,5)		312 (31,1)		181 (30,5)		238 (40,2)		838 (33,0)		
Não informado	48		20		86		107		261		
**Classificação clínica**										<0,001	
Primária	72 (26,0)		121 (12,0)		82 (12,7)		73 (10,5)		348 (13,2)		P1-P2 (<0,001) P1-P3 (<0,001) P1-P4 (<0,001)
Secundária	14 (5,1)		34 (3,4)		8 (1,2)		12 (1,7)		68 (2,6)		
Terciária	119 (43,0)		192 (19,0)		103 (15,9)		118 (17,0)		532 (20,2)		
Latente	72 (26,0)		663 (65,6)		454 (70,2)		491 (70,7)		1.680 (63,9)		
Não informado	122		14		33		5		175		
**Dados laboratoriais**											
**Teste treponêmico no pré**-natal										<0,001	
Não reagente	7 (1,8)		6 (0,6)		45 (6,7)		27 (4,1)		85 (3,1)		P1-P2 (0,002) P1-P3 (<0,001) P1-P4 (<0,001)
Reagente	345 (87,8)		986 (96,4)		590 (87,4)		603 (90,5)		2.524 (91,5)		
Não realizado	41 (10,4)		31 (3,0)		40 (5,9)		36 (5,4)		148 (5,4)		
Não informado	6		1		5		33		45		
**Teste não treponêmico no pré**-natal										<0,001	
Não reagente	34 (9,4)		31 (3,7)		37 (6,2)		25 (3,8)		127 (5,2)		P1-P2 (<0,001) P1-P3 (0,021) P2-P3 (0,015) P2-P4 (<0,001) P3-P4 (0,001)
Reagente	245 (68,1)		504 (60,4)		384 (64,1)		521 (79,1)		1.654 (67,4)		
Não realizado	81 (22,5)		300 (35,9)		178 (29,7)		113 (17,1)		672 (27,4)		
Não informado	39		189		81		40		349		
**Titulação de teste não treponêmico no pré**-natal										0,031	
1:8 a 1:32	252 (77,3)		579 (72,0)		401 (70,0)		460 (76,6)		1.692 (73,4)		-
>1:32 e ≤1:64	64 (19,9)		210 (26,3)		163 (28,4)		131 (21,8)		568 (24,7)		
>1:128	9 (2,8)		14 (1,7)		8 (1,6)		10 (1,7)		41 (1,8)		
**Dados terapêuticos**											
**Tratamento da mulher**										<0,001	
Com penicilina	359 (91,8)		985 (96,4)		622 (92,6)		623 (91,43)		2.589 (93,6)		P2-P3 (0,004) P2-P4 (0,001)
Sem penicilina	6 (1,5)		0 (0,0)		1 (0,1)		4 (0,6)		11 (0,4)		
Não realizado	26 (6,6)		37 (3,6)		49 (7,3)		55 (8,1)		167 (6,0)		
Não informado	9		2		9		15		35		
**Adequação do tratamento^a^**										0,004	
Inadequado	96 (36,4)		149 (15,4)		99 (16,7)		102 (16,5)		446 (18,2)		P1-P2 (<0,001) P1-P3 (<0,001) P1-P4 (<0,001)
Adequado	168 (63,4)		819 (84,6)		494 (83,3)		517 (83,5)		1.998 (81,8)		
Não informado											
**Dados de parceria sexual**											
**Tratamento do parceiro**										0,001	
Não realizado	143 (39,7)		457 (47,8)		278 (49,8)		265 (53,9)		1.143 (48,3)		P1-P2 (0,017) P1-P3 (0,008) P1-P4 (<0,001)
Com penicilina	206 (57,2)		496 (51,8)		274 (49,1)		215 (43,7)		1.191 (50,3)		
Sem penicilina	11 (3,1)		4 (0,4)		6 (1,1)		12 (2,4)		33 (1,4)		
Não informado	39		67		122		207		435		
**Motivo para não tratamento**										<0,001	
Não teve mais contato	72 (46,5)		247 (48,5)		102 (34,3)		89 (25,0)		510 (38,7)		P1-P4 (<0,001) P2-P3 (<0,001) P2-P4 (<0,001) P3-P4 (<0,001)
Não comunicado/convocado à unidade de saúde	2 (1,3)		31 (6,1)		11 (3,7)		8 (2,2)		52 (3,9)		
Não compareceu para tratamento	23 (14,8)		107 (21,0)		56 (18,9)		38 (10,7)		224 (17,0)		
Recusou tratamento	14 (9,0)		11 (2,2)		8 (2,7)		8 (2,2)		41 (3,1)		
Sorologia não reagente	9 (5,8)		34 (6,7)		18 (6,1)		15 (4,2)		76 (5,8)		
Outro motivo	35 (22,6)		79 (15,5)		102 (34,3)		198 (55,6)		414 (1,4)		

^a^Período de desabastecimento de penicilina; ^b^Normalização da produção de penicilina e pré-pandemia de covid-19; ^c^Período de pandemia de covid-19; ^d^Período pós-pandemia de covid-19. O traço indica que nenhuma comparação entre períodos específicos atingiu p-valor>0,005 após a correção com Bonferroni.

A maioria dos testes treponêmicos (91,5%) e não treponêmicos (67,4%) realizados no pré-natal apresentou resultado reagente, com aumento da proporção de resultados reagentes ao longo da série temporal. Observou-se maior frequência de titulações não treponêmicas entre 1/8 e 1/32 (73,4%), sem diferenças entre os períodos analisados (Tabela 3).

A penicilina G benzatina foi prescrita para 93,6% das mulheres, com dose considerada adequada em 81,8% dos casos. Observaram-se redução na prescrição de penicilina (96,4% para 92,6% e 91,4%) e aumento da não realização do tratamento do período pré-pandêmico para os pandêmico e pós-pandêmico (3,6% para 7,3% e 8,1%). A adequação do tratamento apresentou aumento após o período de desabastecimento de penicilina (63,4% para 84,6%, 83,3% e 83,5%) (Tabela 3).

Entre os parceiros sexuais, a prescrição de tratamento não foi realizada em 48,3% dos casos, com aumento progressivo da ausência de tratamento a partir do período de pré-pandemia (39,7% para 47,8%, 49,8% e 53,9%). As principais justificativas para a não prescrição foram a falta de contato com a mulher (38,7%) e outros motivos (31,4%), com diferenças nas proporções entre os períodos analisados (p-valor<0,001) (Tabela 3).

Entre os nascimentos pareados, observou-se predominância de recém-nascidos do sexo masculino (51,3%), a termo (87,2%), com peso adequado ao nascer (84,4%), sem indicação de asfixia no primeiro (95,4%) e quinto (98,9%) minutos de vida e sem anomalias congênitas (99,2%). Nenhuma dessas variáveis apresentou diferenças ao longo do tempo (Tabela 4).

**Tabela 4:** Distribuições absoluta (n) e relativa (%) das características de nascimento das crianças vinculadas às mulheres notificadas com sífilis gestacional segundo os períodos analisados. Campo Grande, 2015-2023 (n=2.227)

Variáveis	2015 a 2016-P1^a^		2017 a 2019-P2^b^		2020 a 2021-P3^c^		2022 a 2023-P4^d^		Total	p-valor
	n (%)		n (%)		n (%)		n (%)		n (%)	
Sexo										0,504
Feminino	160 (50,6)		413 (48,5)		282 (50,6)		234 (46,6)		1.089 (48,9)	
Masculino	156 (49,4)		439 (51,5)		275 (49,4)		268 (53,4)		1.138 (51,3)	
Não informado	83		172		123		197		575	
**Idade gestacional **(semanas)										0,195
Prematuro	44 (13,9)		87 (10,2)		59 (10,6)		70 (13,9)		260 (11,7)	
A termo	267 (84,5)		757 (88,8)		492 (88,5)		426 (84,9)		1.942 (87,2)	
Pós-termo	5 (1,6)		8 (0,9)		5 (0,9)		6 (1,2)		24 (1,1)	
Não informado	83		172		124		197		578	
**Peso ao nascer **(gramas)										0,060
Baixo peso	28 (8,9)		79 (9,3)		75 (13,5)		57 (11,4)		239 (10,7)	
Peso normal	276 (87,3)		724 (85,0)		458 (82,2)		421 (83,9)		1.879 (84,4)	
Macrossômico	12 (3,8)		49 (5,8)		24 (4,3)		24 (4,8)		109 (4,9)	
Não informado	83		172		123		197		575	
**Apgar 1 minuto **(pontos)										0,447
Sem asfixia (≥7)	293 (93,9)		807 (95,3)		534 (95,9)		483 (96,2)		2.117 (95,4)	
Asfixia moderada (4-6)	13 (4,2)		25 (3,0)		12 (2,2)		15 (3,0)		65 (2,9)	
Asfixia grave (≤3)	6 (1,9)		15 (1,8)		11 (2,0)		4 (0,8)		36 (1,6)	
Não informado	87		177		123		197		584	
**Apgar 5 minutos **(pontos)										0,227
Sem asfixia (≥7)	311 (99,7)		835 (98,6)		548 (98,6)		499 (99,4)		2.194 (98,9)	
Asfixia moderada (4-6)	1 (0,3)		6 (0,7)		7 (1,3)		2 (0,2)		16 (0,7)	
Asfixia grave (≤3)	0 (0,0)		6 (0,7)		1 (0,2)		1 (0,2)		8 (0,4)	
Não informado	87		177		125		197		584	
**Presença de anomalia congênita**										0,285
Não	312 (98,7)		846 (99,6)		549 (98,7)		499 (99,6)		2.206 (99,2)	
Sim	4 (1,3)		5 (0,6)		7 (1,3)		2 (0,4)		18 (0,8)	
Não informado	83		173		126		198		578	

^a^Período de desabastecimento de penicilina; ^b^Normalização da produção de penicilina e pré-pandemia de covid-19; ^c^Período de pandemia de covid-19; ^d^Período pós-pandemia de covid-19. O traço indica que nenhuma comparação entre períodos específicos atingiu p-valor>0,005 após a correção com Bonferroni.

No total, 18 recém-nascidos (0,8%) apresentaram anomalias congênitas, e 33,3% apresentaram mais de uma anomalia. Foram identificadas 21 anomalias distintas, das quais 9 (42,9%) corresponderam a malformações e deformidades osteomusculares; 4 (19,0%), a anomalias dos órgãos genitais; 3 (14,3%), a anomalias do olho, ouvido, face e pescoço; 2 (9,5%), a anomalias do aparelho circulatório; 1 (4,8%), à fenda labial e fenda palatina; 1 (4,8%), a anomalias do sistema nervoso; e 1 (4,8%) a anomalias do aparelho respiratório. Devido à baixa frequência, esses dados não foram tabulados.

## Discussão

Neste estudo, a taxa de detecção de sífilis gestacional aumentou de forma expressiva em Campo Grande, com tendência crescente até 2018 e estabilização nos anos seguintes. Apesar dos avanços nas políticas públicas voltadas ao fortalecimento do pré-natal, à ampliação da testagem e à padronização do manejo clínico da sífilis gestacional no Brasil [20-21], a análise da série histórica de uma capital do Centro-Oeste revelou que as fragilidades no diagnóstico oportuno e, sobretudo, no tratamento dos parceiros, aspectos centrais para o controle da doença, ainda persistem. As diferenças observadas entre os períodos sugerem mudanças no perfil assistencial da sífilis gestacional, com melhora do tratamento materno no período pré-pandêmico, alterações no perfil sociodemográfico durante a pandemia e aumento de diagnósticos tardios no período pós-pandêmico.

Este estudo apresentou limitações inerentes ao uso de dados secundários, incluindo subnotificação, erros de preenchimento e comprometimento da qualidade das informações devido a campos ignorados. Observou-se que cerca de 20% dos casos de sífilis gestacional não apresentaram vínculo com o Sinasc, o que pode refletir perdas fetais não registradas ou subnotificação de desfechos adversos. Esses achados indicaram possíveis vieses de informação e reforçaram a necessidade de aprimorar a qualidade do preenchimento e a integração dos sistemas de informação. 

O pareamento manual entre os bancos de dados pode ter introduzido vieses relacionados a erros de classificação, perdas seletivas de registros e variações na qualidade das informações ao longo do tempo, o que pode ter comprometido parcialmente a comparabilidade dos achados. Por fim, a agregação anual dos dados pode ter introduzido viés temporal ao não captar variações intra-anuais e flutuações sazonais, limitando-se à generalização dos resultados e à precisão das estimativas.

A tendência temporal observada em Campo Grande apresentou padrão distinto daquele descrito no Brasil, onde se verificou tendência de crescimento contínuo da taxa de detecção de sífilis gestacional [6,22] de 2014 a 2023 (VPA 11,15; IC95% 2,78; 23,19), assim como nas cinco regiões do país [22]. Ressalta-se que esses estudos não excluíram possíveis casos de cicatriz sorológica, o que pode ter influenciado as estimativas. 

A estabilização observada a partir de 2018, nesta análise, pode estar associada a um conjunto de fatores. Isso inclui melhorias nos serviços de vigilância [23], ampliação do diagnóstico precoce, persistência de lacunas no acompanhamento pré-natal e no tratamento adequado das gestantes e de suas parcerias [3,4], bem como eventos de maior impacto sistêmico, como o desabastecimento de penicilina [7,24] e as restrições de acesso aos serviços de saúde durante a pandemia de covid-19 [10-14]. Assim, a estabilização da taxa de detecção de sífilis gestacional deve ser interpretada com cautela, pois pode refletir tanto avanços na vigilância e na assistência quanto limitações dos serviços que dificultaram a detecção oportuna e favoreceram a subnotificação.

O perfil de maior frequência da doença entre mulheres pardas/pretas/amarelas/indígenas, adultas jovens e sem ocupação foi semelhante ao padrão identificado em Mato Grosso do Sul no período 2008-2018 [25] e no Brasil em 2024 [6,26], onde também se observou concentração dos casos nesses grupos. Esse perfil é consistente com as desigualdades sociodemográficas nacionais, frequentemente relacionadas às barreiras no acesso oportuno aos serviços de saúde [25,27]. 

No Brasil, em análise de dados de 2011 a 2017, em comparação às mulheres brancas, as pardas e as negras apresentaram maior probabilidade de tratamento inadequado (pardas: OR ajustada 1,10; IC95% 1,01; 1,24; negras: OR ajustada 1,23; IC95% 1,07; 1,41), diagnóstico tardio (pardas: OR ajustada 1,13; IC95% 1,08; 1,17; negras: OR ajustada 1,17; IC95% 1,11; 1,24) e menor probabilidade de tratamento do parceiro (pardas: OR ajustada 1,11; IC95% 1,05; 1,17; negras: OR ajustada 1,11; IC95% 1,03; 1,20) [27]. A escolaridade deve ser analisada tanto no contexto da elevação global da escolaridade da população brasileira (2016-2024) [28] quanto no contexto da propagação da sífilis gestacional em diversos cenários socioeconômicos e níveis de desenvolvimento [1,5], ultrapassando grupos tradicionalmente mais vulneráveis [6,26].

Ao longo da série analisada, as características da doença sofreram mudanças pontuais significativas entre os períodos. A partir de 2014, a redução da produção da penicilina aliada a outros problemas logísticos gerou escassez global [7,8] e coincidiu com o aumento de 92% da taxa de detecção de sífilis gestacional em 2017 [6] e com o maior comprometimento do tratamento materno [29]. Na tentativa de atenuar a escassez da medicação, priorizou-se o seu uso para o tratamento de gestantes e de crianças expostas à sífilis [8]. Esse empenho, juntamente com o processo de estabilização, provavelmente contribuiu para a diminuição da prescrição de outras medicações e o aumento da frequência de tratamento adequado no período 2017-2019.

Mesmo com a disponibilidade da medicação, a elevada frequência de não tratamento do parceiro aumenta as chances de reinfecção da gestante, já que o tratamento adequado dela, associado ao tratamento dele, representa redução no risco de sífilis congênita (RR ajustada 0,60; IC95% 0,55;0,66) [4].

De 2017 a 2019, houve a implementação do projeto *Sífilis Não* em Campo Grande. Foi uma estratégia nacional que promoveu o aprimoramento e a ampliação das ações de vigilância, diagnóstico e tratamento da sífilis por meio da qualificação das equipes de saúde, da estruturação dos serviços, da ampliação da testagem, da regularização do fornecimento de penicilina e da redução da transmissão vertical [30]. Entretanto, os achados deste estudo indicaram que, mesmo com a ampliação da capacidade de resposta dos serviços, persistiram falhas no rastreamento da sífilis gestacional, associadas às lacunas no acompanhamento pré-natal e no manejo dos parceiros, e limitações no funcionamento dos serviços, o que pode ter restringido o impacto das ações implementadas. 

Entre 2020 e 2021, a pandemia de covid-19 comprometeu a efetividade das ações de controle da sífilis, em razão da interrupção parcial do funcionamento dos serviços de saúde [10-12]. No Brasil, estimou-se a redução de aproximadamente 2,65 milhões de procedimentos diagnósticos e terapêuticos relacionados à sífilis em 2020, em comparação à média do período 2016-2019, com queda mais acentuada em Mato Grosso do Sul (63%) [12]. Esse contexto pode ter contribuído para reduzir a realização de testes treponêmicos e não treponêmicos e manter as titulações elevadas observadas neste estudo, compatíveis com atraso no diagnóstico e no início do tratamento.

Neste estudo, observou-se a redução de 29% na taxa de detecção de sífilis gestacional entre 2020 e 2021, em contraste com o aumento de 16% observado em nível nacional no mesmo período [6]. O redirecionamento das ações de vigilância para enfrentamento da pandemia, aliado a desafios estruturais dos serviços de saúde, pode ter contribuído para a subnotificação dos casos de maneira geral [31]. Isso indicou que a queda observada reflete mais limitações na detecção e na notificação do que redução real da ocorrência da doença.

A pandemia e as medidas de restrição de contato social intensificaram as desigualdades estruturais e em saúde em todo o mundo [32]. A redução observada neste estudo de casos entre mulheres pretas/pardas/indígenas/amarelas durante esse período pode estar relacionada às barreiras ampliadas no acesso aos serviços de saúde e à detecção dos casos, em um sistema estruturalmente desigual [25]. Evidenciou-se a necessidade de estratégias específicas para reduzir iniquidades no acesso, especialmente em contextos de emergência sanitária. 

Neste estudo, observou-se que, no período pós-pandêmico (2021-2022), a frequência de consultas de pré-natal foi maior se comparada ao período pandêmico. Isso refletiu a retomada do funcionamento dos serviços de saúde e o restabelecimento da rotina assistencial. Essa recuperação pode ter contribuído para estabilizar a tendência da taxa de detecção observada neste estudo, uma vez que o acompanhamento pré-natal adequado favorece o rastreamento, o diagnóstico e o tratamento oportunos da sífilis, reduzindo o risco da infecção durante a gestação [3] e de desfechos neonatais adversos [2-4,9]. 

Apesar do aumento das consultas e início precoce do pré-natal ao longo da série analisada neste estudo, a predominância de diagnósticos tardios (56%) e de fases clínicas avançadas (64%) da sífilis sugere a persistência de falhas no diagnóstico e no tratamento maternos. O atraso no diagnóstico compromete o início oportuno do tratamento, o que aumenta o risco de transmissão vertical e de desfechos neonatais adversos [2-4]. O número de consultas de pré-natal, isoladamente, não é suficiente para prevenir a transmissão vertical quando oportunidades de testagem e de tratamento são perdidas.

Apesar das limitações, no contexto investigado, este estudo permitiu acompanhar as mudanças nas tendências de detecção e analisar variações no perfil de vulnerabilidade e risco ao longo do tempo, fornecendo subsídios relevantes para direcionar políticas públicas. Utilizar o Sinan e o Sinasc fez com que as informações clínicas se ampliassem, além de aumentar a qualidade dos dados, um diferencial a ser destacado. 

A análise detalhada da caracterização clínica-epidemiológica da sífilis gestacional em períodos marcados por eventos de relevância em saúde pública ofereceu visão integrada das mudanças na magnitude da doença e no perfil da população afetada. Os achados reforçaram a necessidade de fortalecimento contínuo da vigilância epidemiológica, com integração efetiva entre os sistemas de informação, qualificação do pré-natal e monitoramento ativo do tratamento das parcerias sexuais, especialmente em cenários de crise sanitária.

A sífilis gestacional apresentou aumento da taxa de detecção até 2018, com estabilização posterior, apesar de avanços no diagnóstico e no tratamento maternos. O fato de que ainda há falhas no tratamento dos parceiros e variações assistenciais reforça a importância de usar evidência epidemiológica para qualificar a gestão e fortalecer políticas públicas de prevenção e controle.

## Data Availability

O banco de dados anonimizado e o roteiro de análise utilizados na pesquisa poderão ser disponibilizados após a publicação sob demanda aos autores mediante solicitação justificada.
